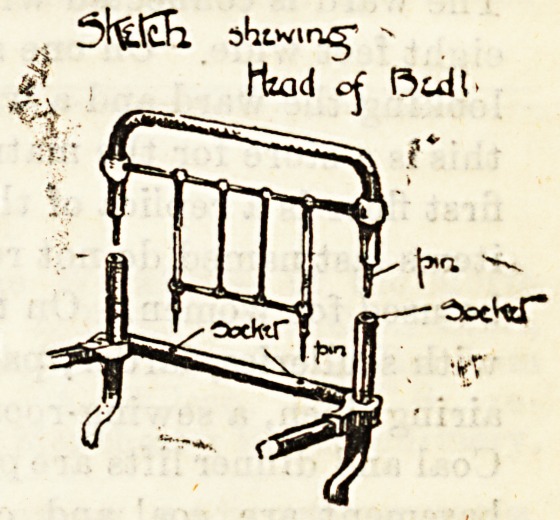# Bedsteads

**Published:** 1894-12-08

**Authors:** 


### PRACTICAL DEPARTMENTS.

### BEDSTEADS.

Perhaps there is no portion of the furniture of the sick room which has undergone more complete and radical change in recent years than the bed itself, to the infinite benefit of patient and nurse alike. We may be thankful indeed for the comfortable and hygienic bedsteads, light and simple in construction, which have taken the place of such atrocities as the erection which ornamented Mrs. Gamp's "apartment" in Kingsgate Street, High Holborn.

With the advent of iron bedsteads the horrors of four posters, "testers," curtains and valance depending to the floor, have gone their ways, and even in lodgings, where such

Hospital Bed.

things have a way of lingering, it is now rare to come upon a real old-fashioned "tent." The number and variety of the shapes and sizes in bedsteads now in use are legion, and it would be hopeless to attempt a description of all those which are excellent in their several ways.

When considering the best pattern and shape for institution use it is naturally even more necessary than in private sickrooms to bear in mind that simplicity of design is essential for purposes of cleanliness and disinfection. Most bedsteads made for hospital use are now very simply constructed and easy to fix, being usually made in three pieces, head and foot, with a frame fitted with galvanised wire springs of varying kinds. Such a bed is the oue shown in our first illustration, which is made by Messrs. Shoolbred, of

Tottenham Court Road, and is excellent in every way for ordinary institution wear.

The height of the bedstead naturally varies; institution authorities, when fitting up wards, usually arranging for the exact dimensions as circumstances render desirable. It is important for the comfort of all concerned that a sick bed should be neither too high for the comfort of the patient when able to get in and out of bed, nor too low for the convenience of the nurse and doctor. A bed should, too, always be high enough to admit of easy sweeping of the floor beneath and the passage of free currents of air. Castors are important features, and need to be strong and steady to allow of easy movement without undue shaking of the patient.

Of beds made for special purposes, many very ingenious inventions may be seen. We give below a sketch of a special bedstead, made by the Longford Company at Warrington, which is in use at the Royal Eye Hospital, Southwark, and is, we believe, the invention of Professor McHardy. The head portion of the bedstead is made in two pieces, the top part fitting into hollow sockets in the lower portion. When it is necessary for an operation to be performed without removing the patient from the bed, the upper part is taken away, and small trays or tables are fitted into the sockets for the convenience of the surgeon.

(*To be continued.*)

## Figures and Tables

**Figure f1:**
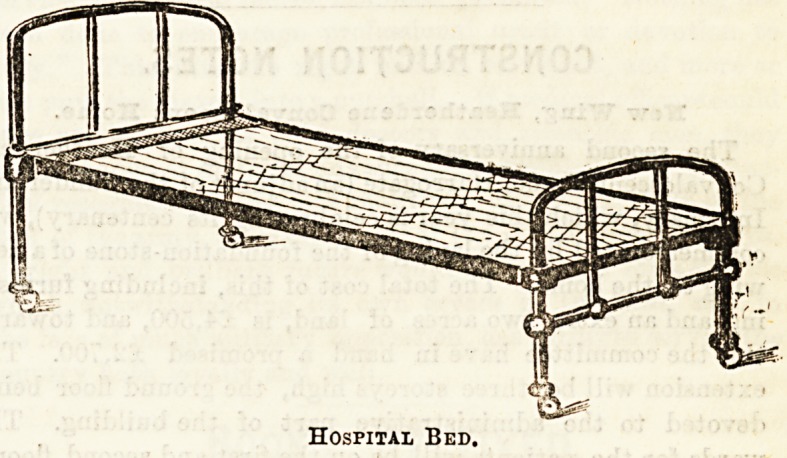


**Figure f2:**
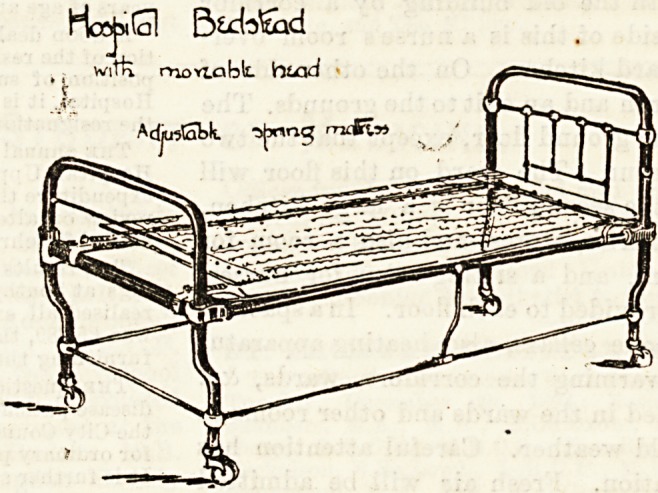


**Figure f3:**